# Gene Expression Pattern of Vacuolar-Iron Transporter-Like (VTL) Genes in Hexaploid Wheat during Metal Stress

**DOI:** 10.3390/plants9020229

**Published:** 2020-02-11

**Authors:** Shivani Sharma, Gazaldeep Kaur, Anil Kumar, Varsha Meena, Hasthi Ram, Jaspreet Kaur, Ajay Kumar Pandey

**Affiliations:** 1 Department of Biotechnology, National Agri-Food Biotechnology Institute, Sector 81, Knowledge City, Mohali, Punjab 140306, India; shivani@nabi.res.in (S.S.); gazaldeep@nabi.res.in (G.K.); anilkumar@nabi.res.in (A.K.); meenavarsha8@nabi.res.in (V.M.); hasthi@nabi.res.in (H.R.); 2 University Institute of Engineering and Technology, Sector 25, Panjab University, Chandigarh, Punjab 160015, India; jaspreet_uiet@pu.ac.in

**Keywords:** micronutrient uptake, *Triticum aestivum* L., Zinc transport, biofortification, Iron deficiency

## Abstract

Iron is one of the important micronutrients that is required for crop productivity and yield-related traits. To address the Fe homeostasis in crop plants, multiple transporters belonging to the category of major facilitator superfamily are being explored. In this direction, earlier vacuolar iron transporters (VITs) have been reported and characterized functionally to address biofortification in cereal crops. In the present study, the identification and characterization of new members of vacuolar iron transporter-like proteins (VTL) was performed in wheat. Phylogenetic distribution demonstrated distinct clustering of the identified *VTL* genes from the previously known *VIT* genes. Our analysis identifies multiple *VTL* genes from hexaploid wheat with the highest number genes localized on chromosome 2. Quantitative expression analysis suggests that most of the *VTL* genes are induced mostly during the Fe surplus condition, thereby reinforcing their role in metal homeostasis. Interestingly, most of the wheat *VTL* genes were also significantly up-regulated in a tissue-specific manner under Zn, Mn and Cu deficiency. Although, no significant changes in expression of wheat *VTL* genes were observed in roots under heavy metals, but *TaVTL2*, *TaVTL3* and *TaVTL5* were upregulated in the presence of cobalt stress. Overall, this work deals with the detailed characterization of wheat *VTL* genes that could provide an important genetic framework for addressing metal homeostasis in bread wheat.

## 1. Introduction

Successful micronutrient biofortification of crops through biotechnology requires detailed knowledge of complex homeostatic mechanisms that tightly regulate the micronutrient concentrations in plants. Iron (Fe) is one of the important micronutrients that is involved in multiple important cellular and physiological processes in plants [1,2,3]. Some of the important functions include its importance in photosynthesis, nitrogen fixation and respiration [4,5]. Although Fe may be present in the soil, yet due to alkaline rhizospheric conditions or unfavorable circumstances, it is not being efficiently taken up by plants [6,7,8,9]. Moreover, the Fe is mobilized through a multistep process that overcomes transport bottlenecks and eventually is loaded in the developing grains [10,11,12,13]. Researchers worldwide are utilizing multiple approaches to either enrich Fe in grains or their storage with enhanced bioavailability [14,15,16,17]. To improve Fe content in cereal grains, multiple transporters and chelators have been targeted through multiple molecular approaches [14,15,18]. A number of additional micronutrient transporters have been identified, those are good candidates for micronutrient biofortification, including transporters belonging to the major facilitator superfamily (MFS) gene family [19,20]. Limited evidences are available that have performed molecular characterization of wheat genes or gene families those which are specifically involved in Fe and Zinc (Zn) homeostasis. Recent reports are emerging for the identification of few functional gene families belonging to, yellow stripe like transporters [21], nicotianamine synthase (NAS), deoxymugineic acid synthase (DMAS) [22], yet many genes families remained to be characterized in hexaploid wheat. Similarly, other wheat genes including Zinc–Induced Facilitator-Like Family (ZIFL) transporters have been characterized for their role in mobilizing the uptake of micronutrient such as Fe and Zn [23]. Most of these gene families are highly upregulated in roots subjected to Fe starvation conditions [24,25]. These works identify some of the important candidate genes as an important resource to strategize approaches for micronutrient biofortification in wheat [26].

Fe storage in seeds gets compartmentalized in major subcellular organelles including chloroplasts and vacuoles. For example, 95% of the iron is stored in vacuoles in the *Arabidopsis* seeds [27]. Vacuoles are an important site for Fe mobilization wherein, they are bound to various chelators like phytic acid, nicotianamine and other organic acids etc. Therefore, uptake of Fe into vacuoles could be an alternate strategy to enhance total micronutrient content with a minimized tradeoff for its toxicity in the tissue. To design such strategy, the role of vacuolar transporters needs to be addressed and exploited [14,28]. Previously, vacuolar iron transporters (VIT) were shown to be play an important role in maintaining Fe in the optimal physiological range and prevent cellular toxicity [14]. *VIT* genes from multiple plant species have been characterized and assessed for their ability to enhance Fe content in cereal crops [15]. These *VIT* genes show high homology with a small family of nodulin like protein containing a CCC-1 (Ca^2+^-Sensitive Cross Complementer) like domain with yeast CCC1p1 [29]. CCC-1 like the domain was initially discovered in yeast encoded for the vacuolar iron transporter in yeast. Furthermore, mutant *ccc1* cells show increased sensitivity to external iron [27,30] *AtVIT1* is one of the early characterized genes showing the presence of CCC-1 like domain and transport of iron to vacuoles [27].

Utilizing the bioinformatics resources, subsequent studies led to the identification of many vacuolar iron transporters-like (VTL) proteins from different plant species. Model species, *Arabidopsis* genome encodes five VTL proteins and overexpression of the few genes have shown increased Fe content in seeds. AtVIT1 protein can transport iron into the vacuoles to counter the toxicity and support the seedling development under enhanced iron conditions [29,31].

Wheat is an important crop that is consumed in many developing countries, including India and is therefore being targeted for trait improvement for nutritional quality. Therefore, the characterization of vacuolar transporters in an important crop such as wheat becomes a prerequisite to address the global issue of biofortification. In the current work genome-wide identification of wheat *VTL* genes was performed. Further, expression studies during different regimes of Fe, Zn and multiple heavy metals was done to gain insight for the regulation of wheat *VTL* genes in a tissue-specific manner.

## 2. Results

### 2.1. Identification, Phylogenetic Analysis and Genomic Distribution of Wheat VTL Genes

**Figure 1:**
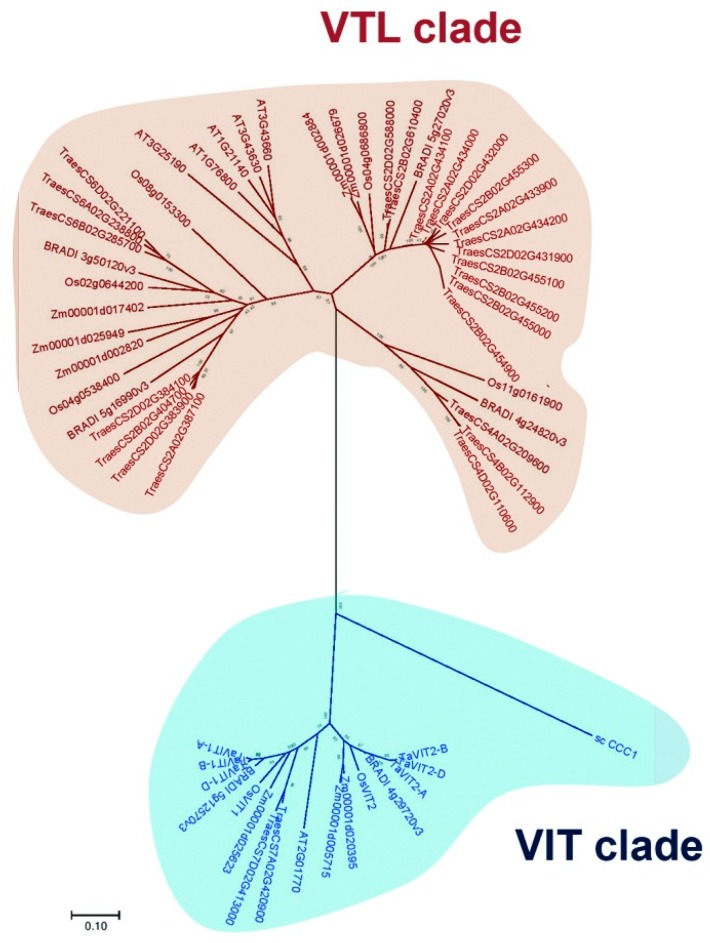
Phylogenetic analysis showing separation of vacuolar iron transporter (VIT) family in *Arabidopsis*, *Brachypodium*, *Oryza sativa*, *Zea mays* and *Triticum aestivum* into two distinct clades; vacuolar iron transporter-like (VTL) clade and VIT clade. The neighbour-joining phylogenetic tree was generated using MEGA. The numbers represent bootstrap values from 1000 replicates.

Thirty-one wheat VIT family sequences were identified based on Ensembl Pfam search and bidirectional BLAST analysis (Table S1). Subsequently, to study the phylogenetic relationship among VIT and VTL family protein sequences from wheat, *Brachypodium*, maize, rice, *Arabidopsis* and *S. cerevisiae*, an unrooted neighbour-joining tree was constructed. This analysis separated the sequences into two distinct clades representing VTL and VIT proteins. This also led to the clustering of the wheat VIT family members into 8 VIT and 23 VTL sequences (Figure 1, Table S2). Due to the occurrence of homoeologs, the 23 VTL sequences were grouped into 4 *VTL* genes and named as *TaVTL1*, *TaVTL2*, *TaVTL4* and *TaVTL5* that corresponds to the rice orthologs followed by the chromosome number. None of the orthologs in wheat showed high confidence similarity with rice vacuolar iron transporter homolog 3. *TaVTL1* and *4* were found to have three homoeologs, while *TaVTL2* had four. In contrast, the phylogenetic analysis grouped 13 highly similar sequences together with rice vacuolar iron transporter homolog 5, these were named as *TaVTL5* (Figure S1).

**Figure 2:**
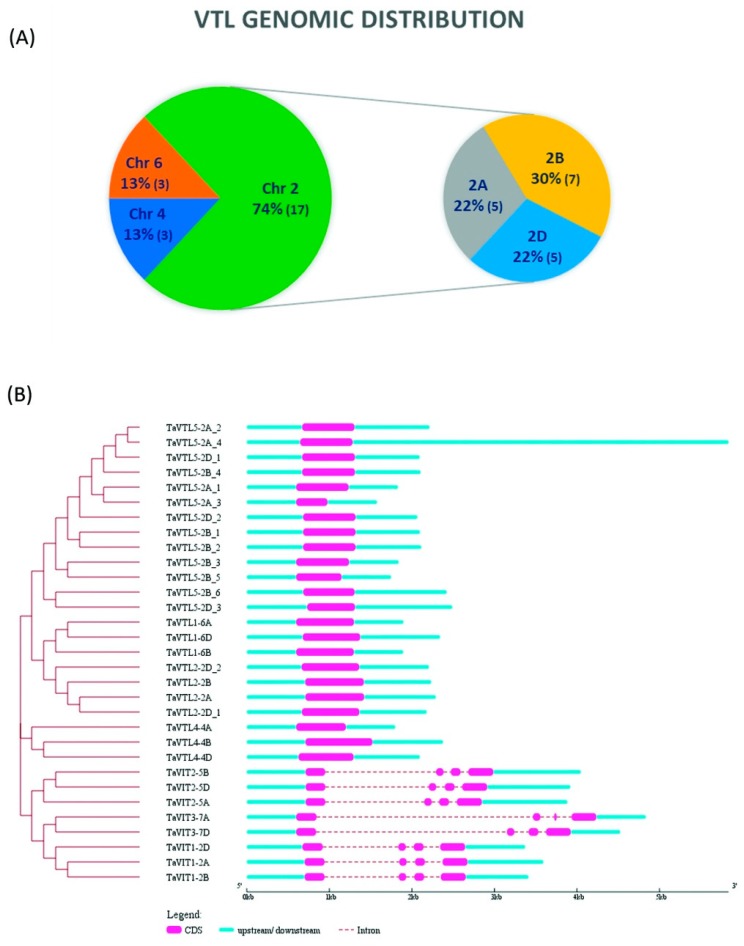
Genomic distribution and exon intron arrangements of *VTL* genes. (**A**) *VTL* genes genomic distribution. Wheat *VTL* genes were present on chromosome groups 2, 4 and 6 with maximum *VTL* genes on chromosome group 2, which was selected to show the *VTL* gene distribution on 2A, 2B and 2D chromosomes. (**B**) Genomic structure for wheat *VTL* and *VIT* genes. The intron-exon arrangement was identified using Gene Structure Display Server (GSDS). Exons and introns are represented using pink boxes and cyan lines, respectively. The scale determines the size of the genomic regions.

*TaVIT1* and *TaVIT2* have already been reported earlier [14]. Interestingly, another new wheat *VIT* with two homoeologs on chromosome 7 (sub-genomes A and D) was identified (referred as *TaVIT3*). *VIT* genes were located on chromosome groups 2, 5 and 7, while *VTL* genes were on chromosome groups 2, 4, and 6 with a maximum contribution from chromosome 2. Nine *VTL* genes were present in the B sub-genome, while seven each on A and D sub-genomes. The maximum number of VTL sequences were located on chromosome 2B (Figure 2A).

### 2.2. Gene, Protein Structure and Subcellular Localization

*VIT* genes in wheat have three and four intronic and exonic regions respectively, while *VTL* genes have a single exon each with the absence of any introns (Figure 2B), clearly dividing the *VIT* family into two sub-families based on gene structure also. CDS length was found to be varying from 657 to 747 nucleotides for wheat *VIT* genes. The CDS length for *VTL* genes was ranging from 549 to 810 nucleotides except for *TaVTL5-2A_3* that was 378 nucleotides long. The short length of one VTL gene is due to the missing sequence information at the stop site. The length of TaVIT peptides ranged from 218 to 256 while TaVTL protein length varied from 125 to 269 amino acids. The division of VIT and VTL proteins was also evident from the sub-cellular localization (Table S2); while TaVIT proteins were predicted to be predominantly localized on the plasma membrane and chloroplast thylakoid membrane, maximum TaVTL proteins were predicted to be present on the vacuolar membrane (87%). TaVTL4-4A was predicted to be localized on plasma membrane. VIT proteins had 3–4 predicted trans-membrane (TM) domains. TaVTL1, 2 and 4 had five TM domains majorly, except for TaVTL4-4B which was predicted to have 6 TM domains. Only TaVTL5-2D_3 had five TM domains; other paralogs/homoeologs of TaVTL5 had lesser number of TM domains probably due to gene duplication events or missing information. To summarize, TaVTLs have five TM domains predominantly, which are depicted in Table S2. VIT1 from *Eucalyptus grandis* (EgVIT1) crystal structure was deciphered recently [32] that was used to confirm the VIT family protein topology prediction using Phobius [33]. EgVIT1 was predicted to have only three TM domains while the crystal structure stated the presence of five TM domains. Therefore, VIT, as well as VTL protein sequences from wheat, were aligned to EgVIT1 to see the possible TM domains in addition to those predicted by Phobius (Figure S2).

### 2.3. Conserved Domain and Motif Analysis

**Figure 3:**
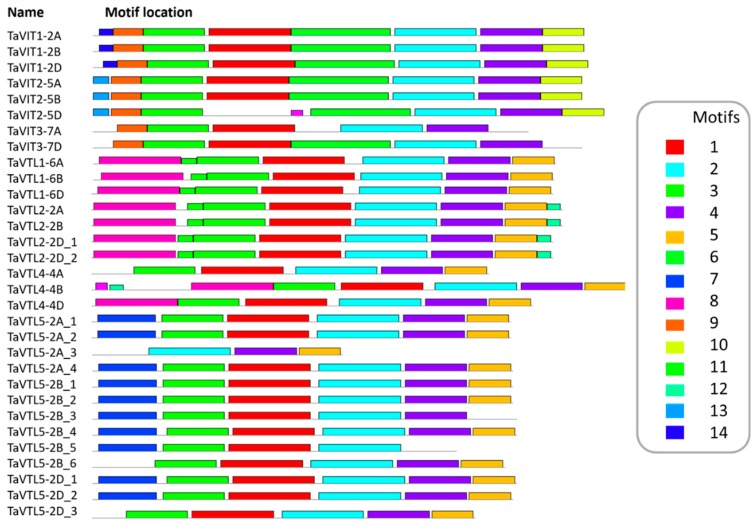
Conserved motifs identified for TaVIT and TaVTL proteins using MEME suite 5.1.0. The colored rectangles on each sequence represent specific conserved motifs numbered 1 through 14, as depicted by the color codes in the box.

All the *VIT* and *VTL* genes were found to have the typical CCC1-like superfamily domains of yeast, which were demonstrated earlier for the iron and manganese transport from the cytosol to vacuole. Motif analysis using MEME webserver suggested that motifs 6, 9, and 10 are VIT specific with exceptions for motif 10 been absent in TaVIT3 and motif 6 absent in TaVIT3-7A. Similarly, motifs 5, 7, 8, 11 to 14 are VTL specific with the exception that motif 5 was absent in TaVTL5-2B_3 and TaVTL5-2B_5, where motif 7 was specific for TaVTL5 sequences except in TaVTL5-2A_3, TaVTL5-2B_6, TaVTL5-2D_3. Motif 8 was specific for TaVTL1, 2 and 4. Motif 11 for TaVTL1 and 2. Motifs 12 and 13 were unique for TaVTL2, whereas Motif 14 was present only in TaVTL1 (Figure 3, Table S3).

### 2.4. Expression of Wheat VTL Genes under Fe Deficiency and Surplus Condition

To check the regulation of *VTL* genes at the transcriptional level, the promoters for the wheat *VTL* genes were scanned for the cis-elements responsive for Fe and heavy metals. The analysis revealed the presence of multiple such sequences, including iron-deficiency-responsive element 1 (IDE1), metal response element (MRE), heavy metal responsive element (HMRE) and iron-related bHLH transcription factor 2 (IRO2) binding site (Table S4). In the most abundant category, iron-deficiency-responsive element 1 (IDE1) was predominant. Interestingly, the IRO2 binding site was present only in the regulatory region of *TaVTL2B/D*. These observations suggest that *VTL* expression could be regulated by the presence-absence of specific metals including micronutrients such as Fe and Zn.

**Figure 4:**
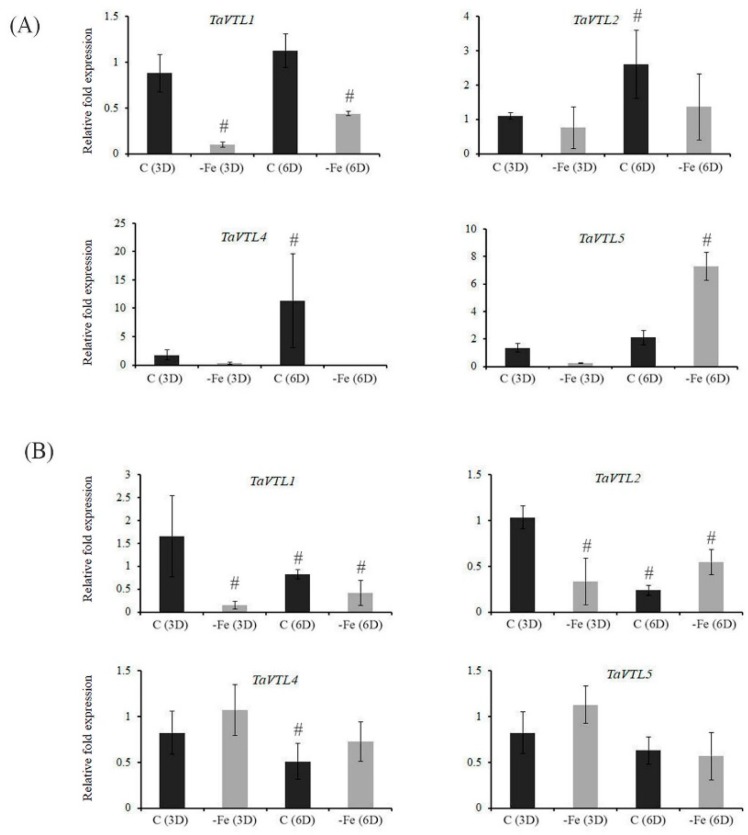
Tissue-specific qRT-PCR expression analysis of wheat VTL genes during Fe deficiency (-Fe) and in the control (C) conditions. Wheat seedlings were subjected to Fe deficiency for three and six days represented as -Fe(3D) and -Fe(6D). The controls for the respective time points are represented as C(3D) and C(6D). (**A**) Fold expression analysis was performed in roots and (**B**) in shoots. 2 µg of total RNA for the cDNA preparation. Relative fold expression levels were calculated relative to C(3D). C_t_ values were normalized using wheat *ARF1* as an internal control. Vertical bars represent the standard deviation. # represents the significant difference at *p* < 0.05 with respect to their respective control treatments.

Previously, *VTL* genes were reported to have differential expression patterns under the changing regimes of Fe and Zn [31]. Therefore, we tested if wheat *VTL* genes could respond at the transcript level when subjected to changing Fe concentration. The expression in roots and shoots of wheat seedlings was measured after subjecting them for three and six days of starvation. Our expression analysis suggests that in roots all the *VTL* genes (*TaVTL1, TaVTL2, TaVTL4* and *TaVTL5*) were downregulated at both the days, whereas, only *TaVTL5* was upregulated at six days of starvation (Figure 4A).

Similarly, in shoots also all the expression of wheat *VTL* genes was suppressed except for *TaVTL2* that was upregulated only on six days post starvation (Figure 4B). These expression data demonstrate that under Fe deprivation *VTL* gene expression are negatively regulated in wheat seedling. Transcriptomic sequencing data from wheat seedlings after 20 days of Fe starvation (SRP189420) were also used to check expression response upon Fe starvation. Categorically, *TaVTL5* group genes were seen to be upregulated upto 12-fold, with *TaVTL5-2B_6* showing upregulation of ~60 fold, although the expression was not very high (Figure S3).

**Figure 5:**
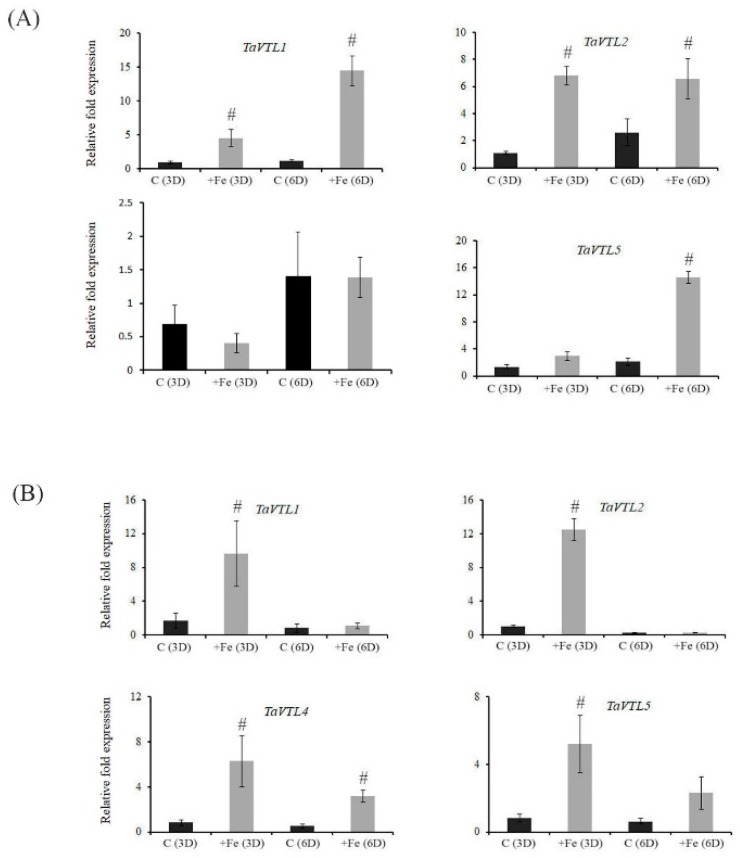
Tissue-specific qRT-PCR expression analysis of wheat VTL genes during Fe surplus (+Fe) and in the control (C) conditions. Wheat seedlings were subjected to Fe surplus for three and six days represented as +Fe(3D) and +Fe(6D). The controls for the respective time points are represented as C(3D) and C(6D). (**A**) Fold expression analysis was performed in roots and (**B**) in shoots. 2 µg of total RNA for the cDNA preparation and relative fold expression levels were calculated relative to C(3D). C_t_ values were normalized using wheat *ARF1* as an internal control. Vertical bars represent the standard deviation. # represents the significant difference at *p* < 0.05 with respect to their respective control treatments.

Next, we performed the gene expression analysis under the excess Fe regime. This was done to test if wheat *VTL* genes could be potentially involved in detoxification of excess Fe. Interestingly, we observed a significant up-regulation of all the *TaVTL* genes in roots at both the time points. Out of all, *TaVTL4* showed the highest fold gene expression (~100 fold) when compared to its control (Figure 5A).

*TaVTL2* show very early and high expression response, whereas both *TaVTL1* and *TaVTL5* were highly expressed at six days of treatment. At this time their gene expression level was more than ~14 fold compared to control. In contrast, in shoots most of the wheat *VTL* genes were expressed at the three days of treatment with *TaVTL1* and *TaVTL2* showing the transcript accumulation of 8–14 folds with respect to their control (Figure 5B).

### 2.5. Manganese, Zinc and Copper Deficiency Causes Differential Changes in VTL Expression

**Figure 6:**
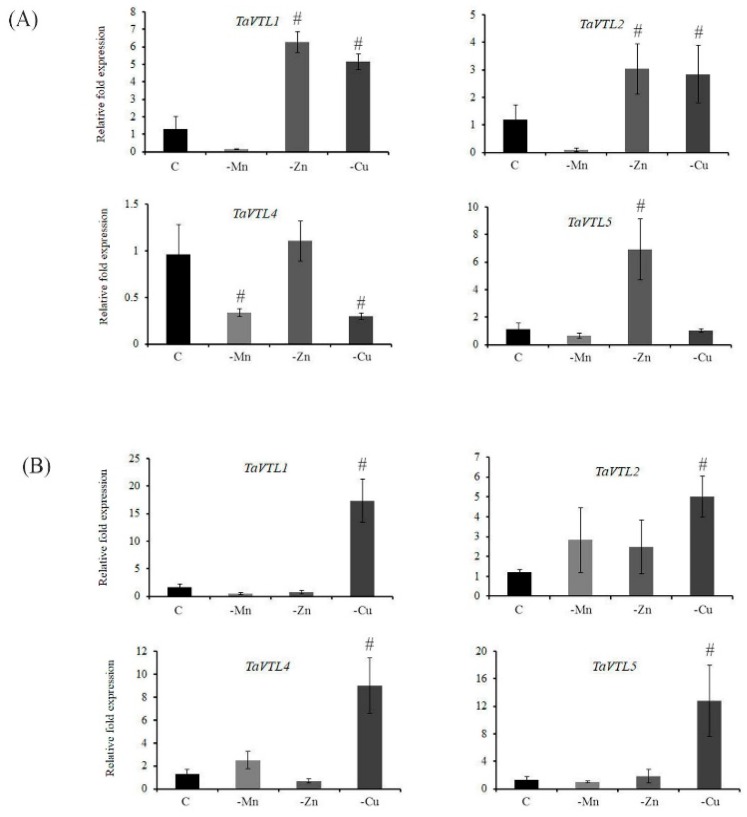
Tissue-specific qRT-PCR expression analysis of wheat *VTL* genes during Mn (-Mn), Zn (-Zn) and Cu (-Cu) deficiency with respect to the control (C) conditions. (**A**) Fold expression analysis was performed in roots and (**B**) in shoots. 2 µg of total RNA was used for the cDNA preparation and relative fold expression levels were calculated relative to control tissue (C). The C_t_ values were normalized using wheat *ARF1* as an internal control. Vertical bars represent the standard deviation. # represents the significant difference at *p* < 0.05 with respect to Control tissue.

Wheat *VTL* genes showed high similarity to previously known *VIT* genes. In addition to Fe, *VIT* genes are known to be affected by the perturbed concentration of Mn [14]. Since many of these cation transporters are known for their reduced substrate specificity [34,35], therefore, expression of wheat *VTL* genes during Zn, Cu and Mn deprivation was also studied (Figure S4A). In general, during the changing regimes of Zn and Mn, wheat *VTL* genes showed specific expression in a tissue-specific manner (Figure 6). *TaVTL2* was the only gene showing enhanced accumulation of its transcript under Zn deficiency in both root and shoot tissue, whereas *TaVTL1* and *TaVTL5* showed high expression in roots only under Zn deficiency (Figure 6A and B). No significant changes in the expression of *TaVTL4* were observed for the studied time point under the changing Zn regime. In contrast, no induction of wheat *VTL* genes was observed in roots under Mn deficiency with respect to its control, whereas, in shoots, *TaVTL2* and *TaVTL4* showed high transcript accumulation (Figure 6A). Under Cu deficiency, all *VTL* genes showed an induced expression in shoots while only two of the genes, including *TaVTL1* and *TaVTL2* were upregulated in roots.

### 2.6. Heavy Metal (Ni, Cd and Co) Mediated Expression of VTL Genes

**Figure 7:**
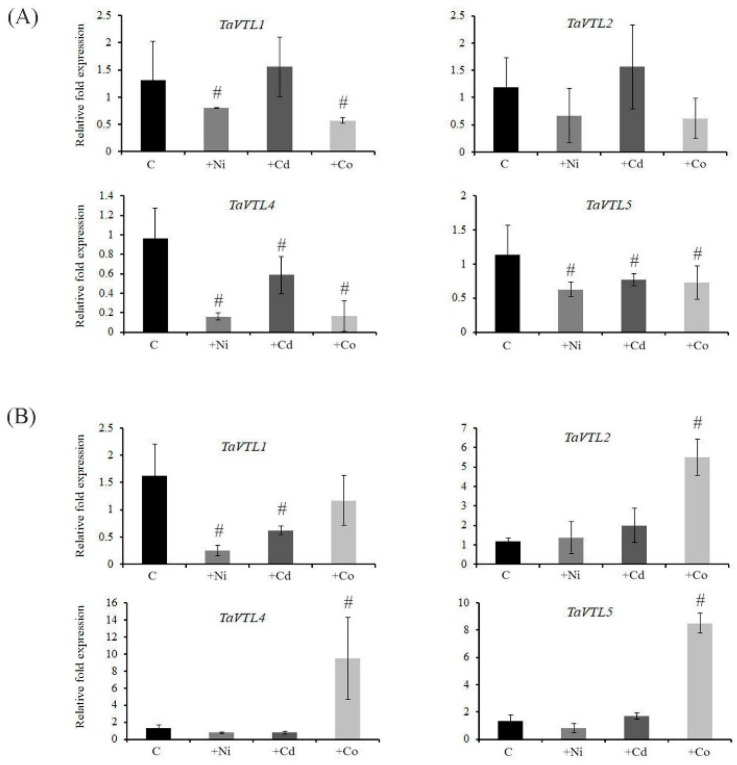
Tissue-specific qRT-PCR expression analysis of wheat *VTL* genes upon heavy metal treatments. Wheat seedlings were exposed to Ni (+Ni, 50 µm), Cd (+Cd, 50 µm) and Co (+Co, 50 µm). Control seedlings (C) without any exposure to heavy metals were compared with the treated ones. (**A**) Fold expression analysis was performed in roots and (**B**) in shoots. 2 µg of total RNA for the cDNA preparation and relative fold expression levels were calculated relative to control samples. C_t_ values were normalized using wheat *ARF1* as an internal control. Vertical bars represent the standard deviation. # represents the significant difference at *p* < 0.05 with respect to their respective control treatments.

To check the effect of the heavy metal stress on the gene expression pattern, wheat seedlings were subjected to treatment with Ni, Cd and Co and expression of *VTL* genes was performed. In the treated plants, decreased growth of the shoot and root length was observed, suggesting that heavy metals could affect the plant performance (Figure S4A). In general, the presence of heavy metals led to significant retardation in the growth of roots and shoots, thereby impacting the total plant growth (Figure S4B). Interestingly, none of the wheat *VTL* genes showed enhanced expression in roots after 15 days of heavy metal exposure, but downregulation was observed for *TaVTL1*, *TaVTL4* and *TaVTL5* (Figure 7A). In shoots, only Co stress could influence the gene expression when compared to the control. Only, *TaVTL2*, *TaVTL4* and *TaVTL5* genes were upregulated during the Co stress as compared to control shoot samples (Figure 7B). Altogether, this suggests the metal-specific expression of *VTL* genes in a tissue-specific manner. The previously reported wheat *VIT* genes showed grain specific expression data. Surprisingly, *VTL* genes showed very low or no expression in grains or their tissue parts, suggesting their probable roles in the specific organs of the plants (Figure S5).

## 3. Discussion

Fluctuation in the nutrient availability in the soil results in suitable adaptations by the plants. In general, plants rely on different physiological and molecular processes to minimize nutrient stress [36]. In this regard, MFS gene family plays an important role to provide the tolerance as well as mobilization of important minerals, including micronutrient translocation to the foliar parts including seeds [19]. In this study, the characterization of VTL was done in the hexaploid wheat. Our data reinforce the importance of *VTL* genes for their roles during metal homeostasis and substantiated them as a good candidate for micronutrient biofortification in cereal crops such as wheat and rice.

MFS family has been widely explored for its role as metal transporters and providing the necessary support for multiple functions in plants [37]. Previously, five *VTL* genes were reported in *Arabidopsis* and rice for this sub-class. Our study in wheat resulted in the identification of a maximum number of *VTL* genes from any crop plants. The high number of genes is due to the presence of multiple homoeologous and occurrence of the duplication of multiple wheat *VTL* genes. Interestingly, chromosome 2 having the highest number of wheat *VTL* genes has been linked with multiple quantitative trait loci (QTL) for the high grain Fe and Zn content [38]. Further dissection is required in this direction to identify if any of the wheat VTL could be linked with the loading of micronutrient in grains. Based on our expression analysis and the support from the previous studies, it could be suggested that *VTL* genes could also be involved in providing the tolerance to high levels of Fe and Zn in the soils [31]. In fact, the predicted localization data indicate that VTL could be localized at either the plasma membrane or the vacuolar membrane (Table S2). AtVTL1 was reported to be localized in the vacuolar membrane and others been associated with the plasma membrane [31]. Our PSORT analysis suggests that most of the wheat VTL proteins are localized in the vacuolar membrane, thus making them a suitable candidate for sequestering micronutrients such as Fe and Zn. AtVTL1 also rescued Δccc1 function in yeast by catalysing Fe uptake [31]. Vacuoles are the prime sites for the sequestration of micronutrients such as Fe. Whether, any of these predicted vacuolar TaVTL proteins could perform similar function as TaVIT2 for Fe biofortification needs to be studied [14].

The substrate specificity of the metal transporters is a major bottleneck to achieve high Fe and Zn in grains. Manipulating the specificity of these metal transporters to enrich the Fe and Zn remains the major challenge [23,34,39]. Therefore, studying the expression pattern of *VTL* genes in the presence of heavy metals could provide preliminary clues for employing such strategies. Consequently, the study was undertaken to see the influence of other metals like Ni, Cd and Co. The expression of wheat *VTL* genes in roots and shoots suggested an interesting phenomenon, where no significant changes in the expression of their transcript was observed when exposed to either Ni or Cd. In contrast, only Co was able to induce the expression of *TaVTL1*, *TaVTL2*, *TaVTL4* and *TaVTL5* in shoots only (Figure 7B). These data suggest the controlled expression of wheat *VTL* genes in a tissue specific manner. Additionally, besides Fe homeostasis, the vacuolar transporters are also linked with the impaired activity of Zn and Mn transport [39]. In our study only, *TaVTL2* was significantly induced by the Mn deficiency in shoots (Figure 6B). No such effects were observed in roots wherein, all the quantified wheat VTL showed downregulation under Mn deficiency (Figure 6A). Interestingly, *TaVTL1* and *TaVTL2* showed upregulation in roots under Zn deficiency (Figure 6A). The tissue dependent expression patterns of wheat *VTL* genes under the changing regimes of the metal exposure was observed. It has been observed that *VTL* genes from *Arabidopsis* showed transcriptional changes in response to Fe, Zn and Mn [31]. Based on the previous work and our results it could be suggested that regulation of the *VTL* genes at the transcript level could be conserved. Infact, wheat *VIT* genes can also transport Mn and Fe [14]. This suggest that *VTL/VIT* genes could be regulated only by Fe but also by other metals like Mn and Zn. Additionally, our gene expression data also corelate with the presence of multiple cis-elements in the promoter of wheat *VTL* genes. This indicated that primarily *VTL* genes could be involved during metal homeostasis related responses. Coupled with the localization information, it is possible that few wheat VTL proteins could sequester metals in an organelle specific manner.

Herein, a detailed inventory, structure and expression characterization of wheat *VTL* genes was performed. The expression analysis and analysis for the cis-elements in the promoters of wheat *VTL* genes implicated for their role in metals homeostasis including in Fe and Zn. Overall, the work presented here provide an important framework for identifying the molecular and physiological functions in bread wheat.

## 4. Materials and Methods

### 4.1. Plant Materials and Growth Conditions

For stress experiments, hexaploid wheat *Triticum aestivum* cv. C-306 (received from Punjab Agriculture University, Ludhiana) was used. Briefly, seeds were surface sterilized using 1.2% sodium hypochlorite prepared in 10% ethanol and then rinsed twice with autoclaved MQ. The seeds were kept on moist filter paper inside a Petri dish and stratified for 1 day at 4 °C in dark condition. Stratified seeds were further kept for germination for six days at room temperature. The remaining seed/endosperm were excised from seedlings at one leaf stage and was shifted to phytaboxes (10–12 seedling/phytabox) containing the Hoagland nutrient media for respective treatments. The standard composition of nutrient media for control includes 6 mM KNO3, 1 mM MgSO_4_, 2 mM Ca(NO3), 2 mM NH_4_H_2_2PO_4_, 20 µM Fe-EDTA, 25 µM H3BO3, 2 µM MnSO4, 0.5 µM CuSO4, 2 µM ZnSO4, 50 µM KCl and 0.5 µM Na2MoO4. The variable concentrations used for treatments were excess Fe (+Fe; 200 µm), Fe starvation (-Fe; 2 µm), Zn deficiency (-ZnSO4; 0 µm), Mn deficiency (-MnSO4; 0 µm), Cu deficiency (-CuSO4; 0 µm), Cadmium stress (+Cd; 50 µm) [40], Cobalt stress (+Co; 50 µm) [41] and Nickel stress (+Ni; 50 µm) [42]. The aerobic condition was provided in hydroponics and the media was replaced every alternate day to avoid any contamination and drastic nutrient depletion. The respective roots and shoots samples belonging to iron deficient and sufficient plant groups were collected at three and six days after stress (D). For the rest of the treatments, root and shoot, samples were collected on the 15th Day of treatments. All the experiments were performed in a growth chamber under controlled environmental conditions at 22–24 °C temperature, 65%–70% humidity, at a photoperiod of 16 h day and 8 h night and 300 nm of light.

### 4.2. Identification of VIT Family and Classification of VTL Genes in Wheat

For the identification of wheat *VTL* genes, the Ensembl database was used to extract *VIT* family genes (Pfam ID: PF01988) for wheat. The identification was confirmed by bidirectional BLAST analysis. VIT family sequences from Arabidopsis, rice, maize, *Brachypodium* were also extracted using Pfam search. The identity of VIT family genes was further validated by confirming the presence of CCC1-like superfamily domain using NCBI-CDD domain search. CCC1 sequence for *S. cerevisiae* was also retrieved from its genome database. To separate out *VTL* genes from *VIT* genes and for further phylogenetic analysis, all the proteins were aligned through MUSCLE alignment and an unrooted neighbor-joining phylogenetic tree with 1000 bootstrap replicates was constructed with all the retrieved sequences. The tree was constructed through MEGA-7 [43]. Rice vacuolar iron transporter homolog 1–5 from UniProt were used for the nomenclature of the 23 *TaVTL* sequences based on the closest orthologs. The naming of the genes indicates the chromosome number and the sub-genome on which they are present.

### 4.3. Conserved Domains and Motif Detection, Analysis of Gene, Promoter and Protein Structure

Wheat VIT family genes were searched for conserved domains using NCBI-CDD database [44]. MEME suite v5.1.0 was used for further analysis to identify the common conserved motifs for both VIT and VTL proteins. The maximum number of motifs was set to 15 for MEME analysis. Gene structure for VITs and VTLs was studied using (GSDS) (http://gsds.cbi.pku.edu.cn/) [45] using genomic and CDS sequences. Sub-cellular localization and TM domains were predicted using web-based prediction programs Wolf PSORT and Phobius respectively [46]. For promoter analysis, ~2 Kb promoter elements of the corresponding wheat *VTL* genes were surveyed for the presence of the respective cis-elements. The promoter sequence was obtained for the respective genes using the IWGSC

### 4.4. Total RNA Isolation and cDNA Preparation

The collected root and shoot samples were ground separately in liquid nitrogen. Total RNA from respective samples was extracted by TRIZOL based method. The extraction was followed by the DNase treatment using Turbo DNAfree kit (Invitrogen, Carlsbad, CA, USA) to remove any genomic DNA contamination in the RNA samples. Subsequently, RNA purity was checked and quantified for the preparation of the cDNA. 2 μg of total RNA was used for cDNA synthesis using superScript III First-Strand Synthesis System (Invitrogen, Carlsbad, CA, USA). The cDNA quality was ascertained by using internal control and was further diluted 20X and used for gene expression studies.

### 4.5. Quantitative-Real Time PCR (qRT-PCR) Expression Analysis

To perform quantitative real time-PCR (qRT-PCR), forward and reverse primers of *TaVTL* genes were designed and used as listed in Table S5. The primers were designed from the conserved region of the all homoeolog of each gene. For *TaVTL5* the primers were designed from the conserved region of nine sequences, the significant conserved region was not found for remaining four homoeologs (TraesCS2B02G454900, TraesCS2B02G610400, TraesCS2D02G431900, TraesCS2D02G588000). qRT-PCR was performed in 7500 Real-Time PCR System (Applied Biosystems, Foster City, CA, USA) using 1/20 times dilution of the respective cDNAs. All qRT-PCR reactions were performed using SYBR Green I (QuantiFast^®^ SYBR^®^ Green PCR Kit, Qiagen, Hilden, Germany) chemistry and ARF (ADP-Ribosylation Factor: *TaARF1*—AB050957.1) as an internal control [40]. The efficiency of the qRT-PCR was checked and melt curve analysis was performed for each of the PCR reactions as per the guidelines. Gene expression analyses was carried out with three biological replicates and 2–3 technical replicates. Relative fold expression of genes was determined based on delta-delta CT-method (2^ΔΔCT^) [47].

### 4.6. RNA-Seq Expression Analysis for VIT Family Genes

To get the transcript expression levels for VIT family genes under Fe stress, RNAseq data from SRA project ID SRP189420 were utilized to extract transcript expression values (as FPKM) from control as well as Fe starved wheat root samples using the cufflinks pipeline. Subsequently, for expression analysis of *VTL* and *VIT* genes in wheat grain tissue developmental time course [48], expression values as Transcripts Per Kilobase Million (TPM) were retrieved from expVIP database [49]. Expression values from both studies were then used to plot heatmaps using MeV software (mev.tm4.org).

### 4.7. Statistical Analysis

Excel was used for data analysis. The mean values were calculated form the standard deviation including three technical replicates from at least three biological replicates. Student *t*-tests were used to observe the significant differences between the mean values of treatment and control plants. The significance threshold used was ^#^*p* < 0.05.

## 5. Conclusions

The present work led to the identification of high number of *VTL* genes from hexaploid wheat. Because of polyploidization, a very high number of genes from this sub-family was identified. The presence of high number of VTL been restricted to only chromosome 2, 4 and 6 of the wheat genomes. The expression of these gene under metal stress including changes in the presence of Fe and Zn concentrations and exposure to heavy metals reinforce the importance of this gene-family during metal homeostasis. Our work will help in better understanding of the Fe transporters significance in metal homeostasis so as to biofortify wheat.

## Supplementary Materials

The following are available online at https://www.mdpi.com/2223-7747/9/2/229/s1, **Table S1**: List of 31 VIT family genes extracted from ensembl biomart using Pfam ID: PF01988. **Table S2**: Subcellular localization (WolfPsort). **Table S3**: Conserved motifs identified in VTL and VIT proteins using MEME suite. The color code, consensus sequence logo, E-value and the number of proteins in which each motif was found are listed in the table. **Table S4**: Metal-responsive cis-elements found in *VTL* and *VIT* gene promoter regions. **Table S5**: List of gene specific primers used for qRT-PCR for *TaVTL* genes. **Figu****re S1**: Phylogenetic tree for VIT family genes from *Arabidopsis*, *Brachypodium*, *Oryza sativa*, *Zea mays* and *Triticum aestivum.* Sequences were extracted using Pfam ID followed by alignment by Muscle and construction of NJ tree using MEGA software. **Figure S2**: Transmembrane domains in wheat VIT family proteins. Figure shows potential TM domains in *TaVIT* and *TaVTL* proteins, based on the alignment with *EgVIT1* protein, using MUSCLE. **Figure S3**: Heatmap depicting the expression of VIT family genes (VIT and VTL genes) in Control (FPKM_Control) and Fe starved (FPKM_Fe) wheat roots. FPKM values were extracted using Cufflinks pipeline from SRA projectID SRP189420. Increasing intensity of blue colour shows increase in expression as shown by the colour bar above. **Figure S4**: Effect of different metals on the phenotype and growth of wheat seedlings. (A) Phenotype of wheat seedlings showing retarded growth of shoots and roots. (B) Impact of different metals on the growth (in cm) of roots and shoots. **Figure S5**: Heatmap depicting the expression of VIT family genes (VIT and VTL genes) in Control (FPKM_Control) and Fe starved (FPKM_Fe) wheat roots. FPKM values were extracted using Cufflinks pipeline from SRA project ID SRP189420. Increasing intensity of blue colour shows increase in expression as shown by the colour bar above.
