# Supplementary material for: Gene Expression Pattern of Vacuolar-Iron Transporter-Like (VTL) Genes in Hexaploid Wheat during Metal Stress
Source: Plants (Basel). 2020 Feb 11;9(2):229. doi: 10.3390/plants9020229 (PMC7076494; doi:10.3390/plants9020229)
Supplement: Supplementary file 1 [file plants-09-00229-s001.pdf]

plants

MDPI

Supplementary Material

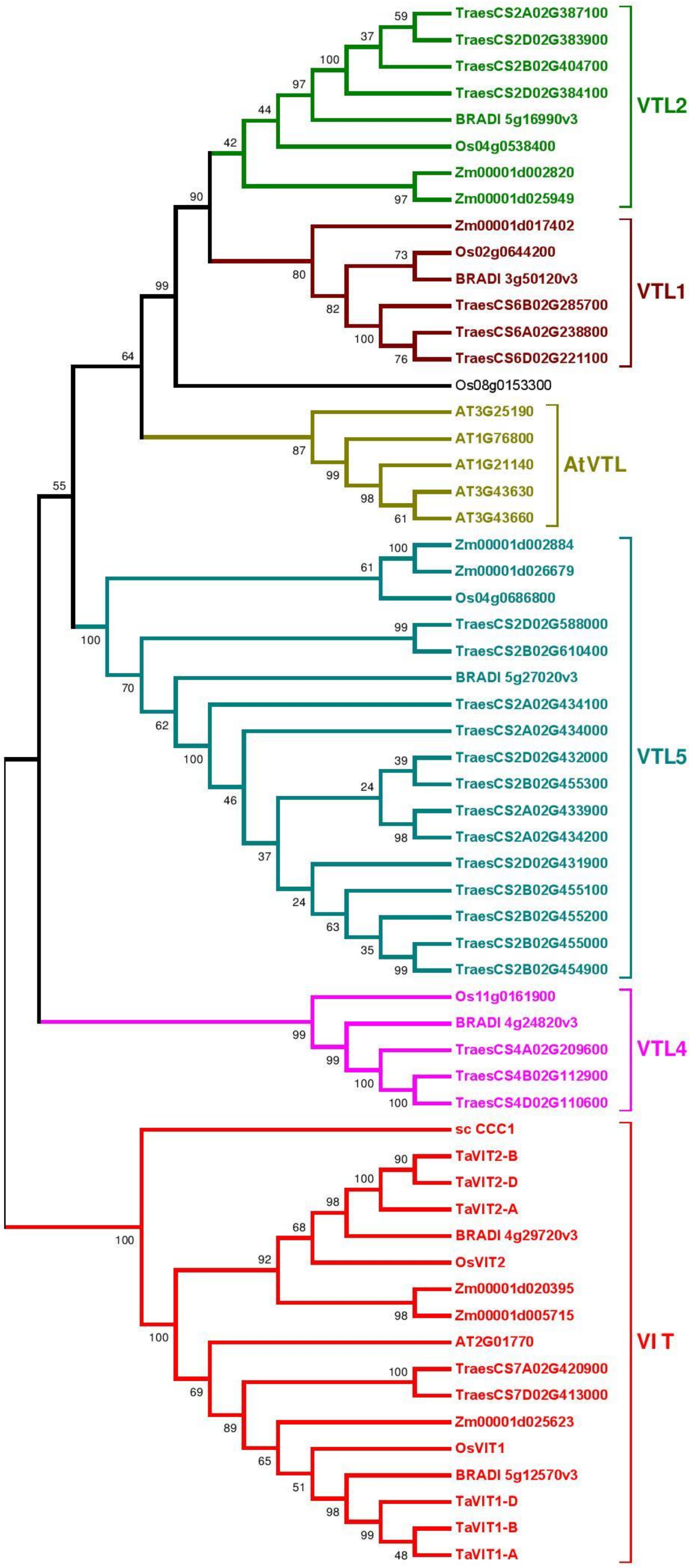

Figure S1. Phylogenetic tree for VIT family genes from *Arabidopsis*, *Brachypodium*, *Oryza sativa*, *Zea mays* and *Triticum aestivum*. Sequences were extracted using Pfam ID followed by alignment by Muscle and construction of NJ tree using MEGA software.

.

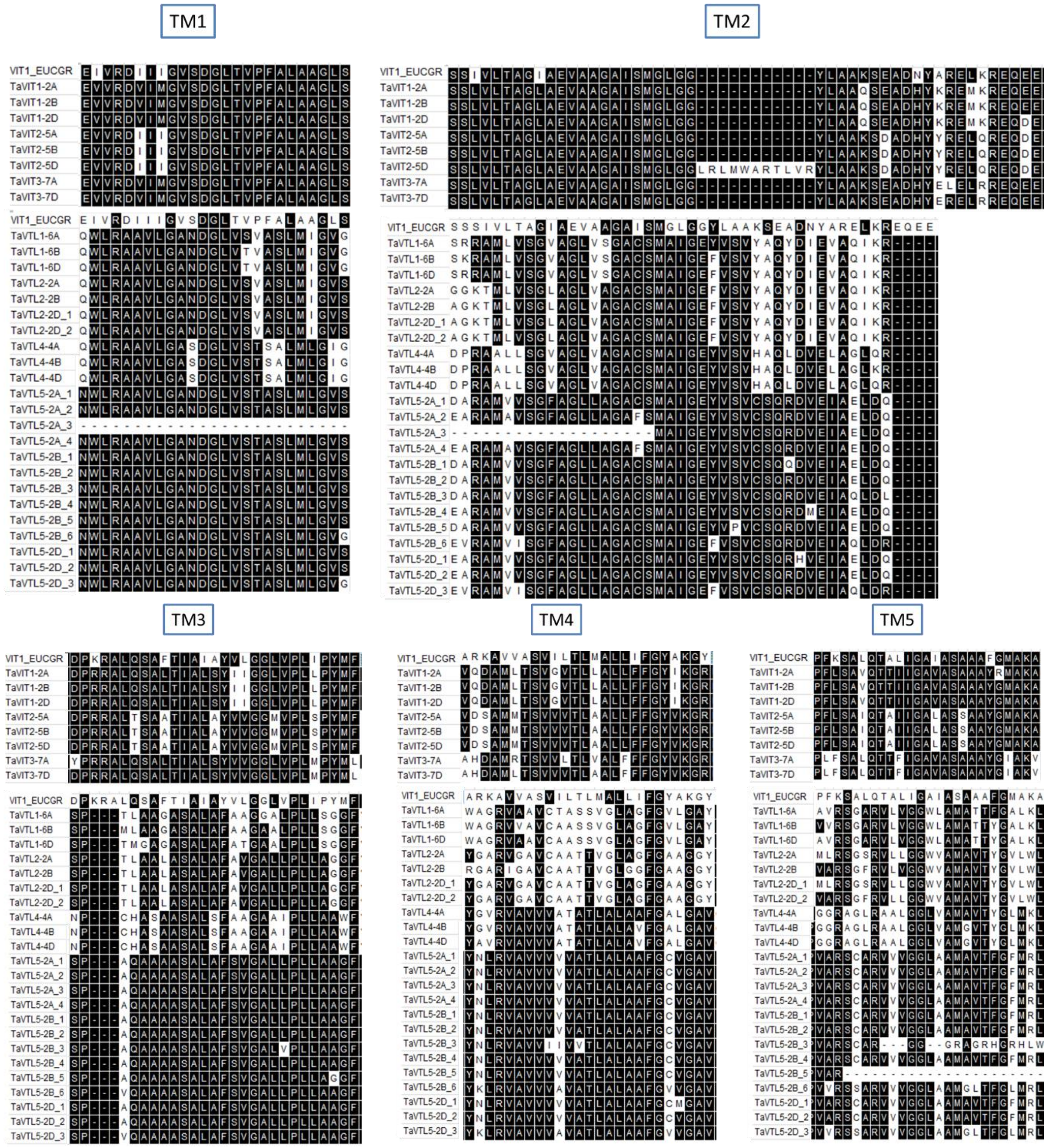

Figure S2. Transmembrane domains in wheat VIT family proteins. Figure shows potential TM domains in *TaVIT* and *TaVTL* proteins, based on the alignment with *EgVIT1* protein, using MUSCLE.

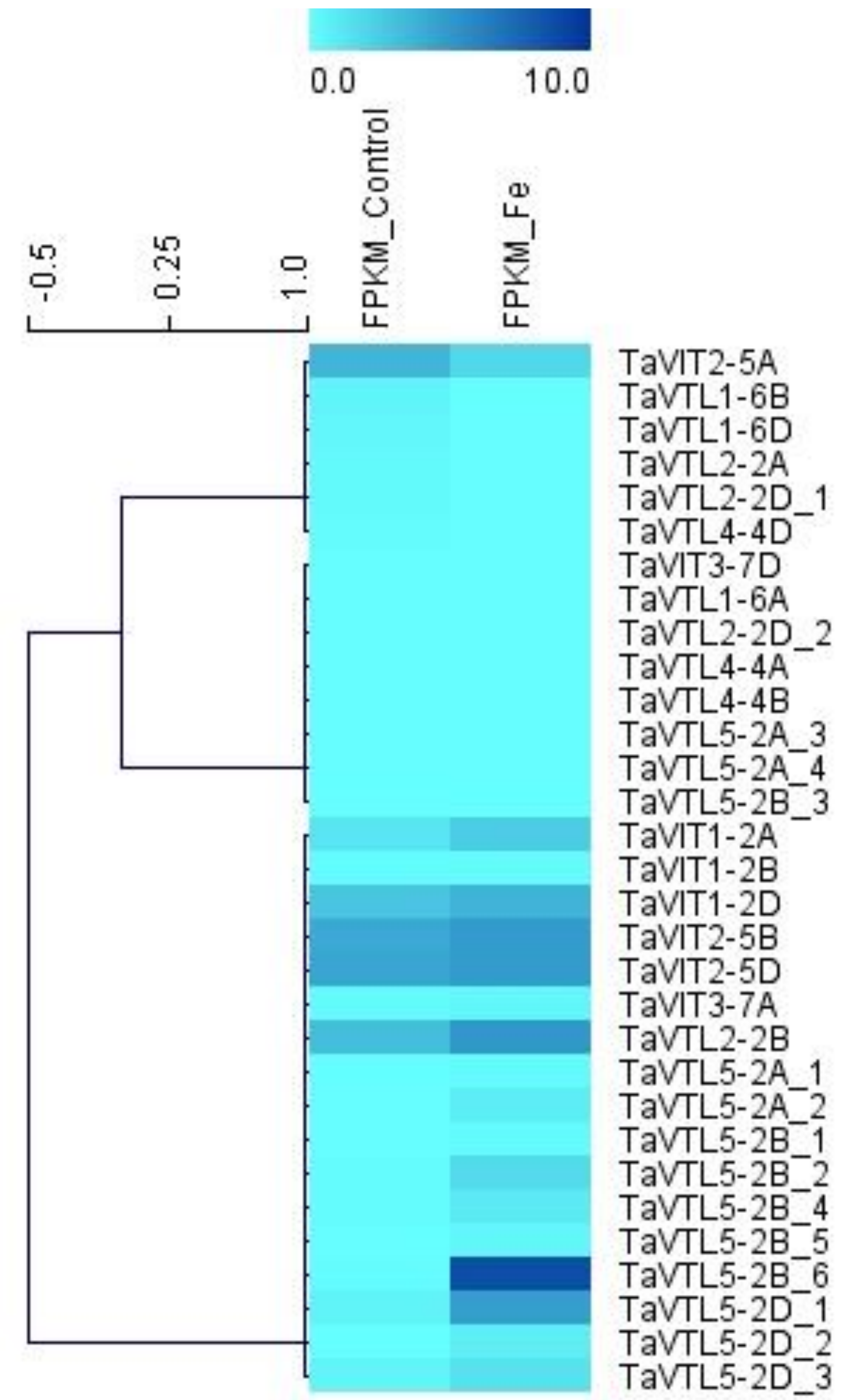

Figure S3. Heatmap depicting the expression of VIT family genes (VIT and VTL genes) in Control (FPKM_Control) and Fe starved (FPKM_Fe) wheat roots. FPKM values were extracted using Cufflinks pipeline from SRA projectID SRP189420. Increasing intensity of blue colour shows increase in expression as shown by the colour bar above.

(A)

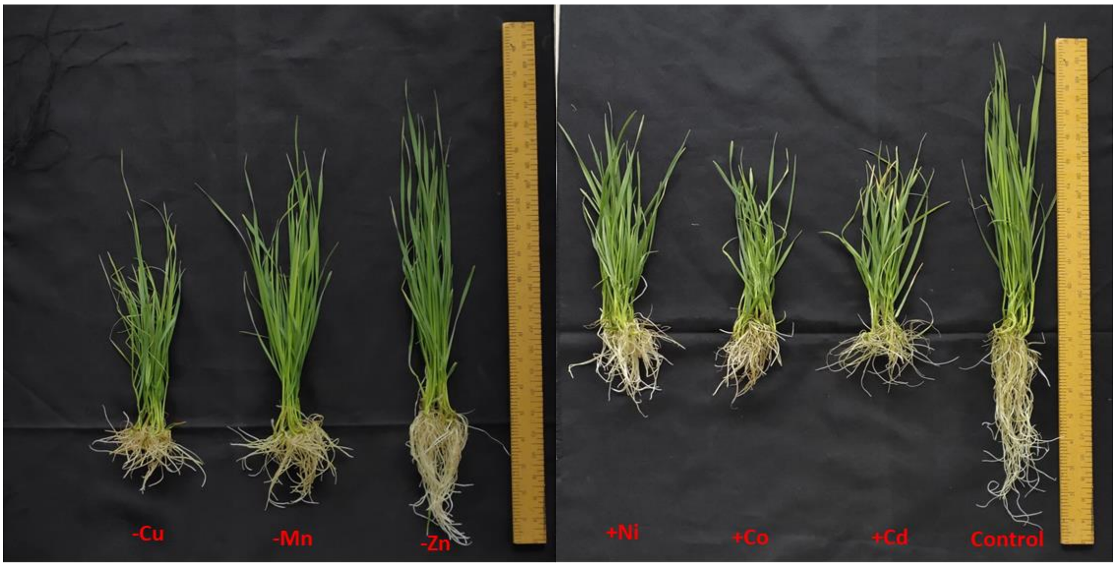

(B)

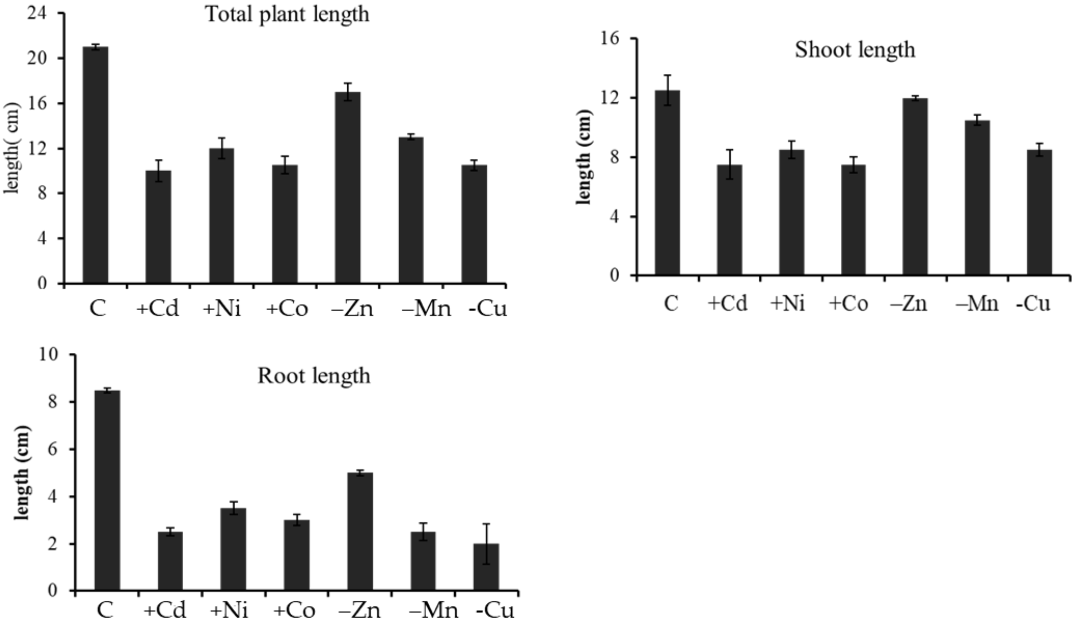

Figure S4. Effect of different metals on the phenotype and growth of wheat seedlings. (A) Phenotype of wheat seedlings showing retarded growth of shoots and roots. (B) Impact of different metals on the growth (in cm) of roots and shoots. Details of the experiments are mentioned in Materials and methods section.

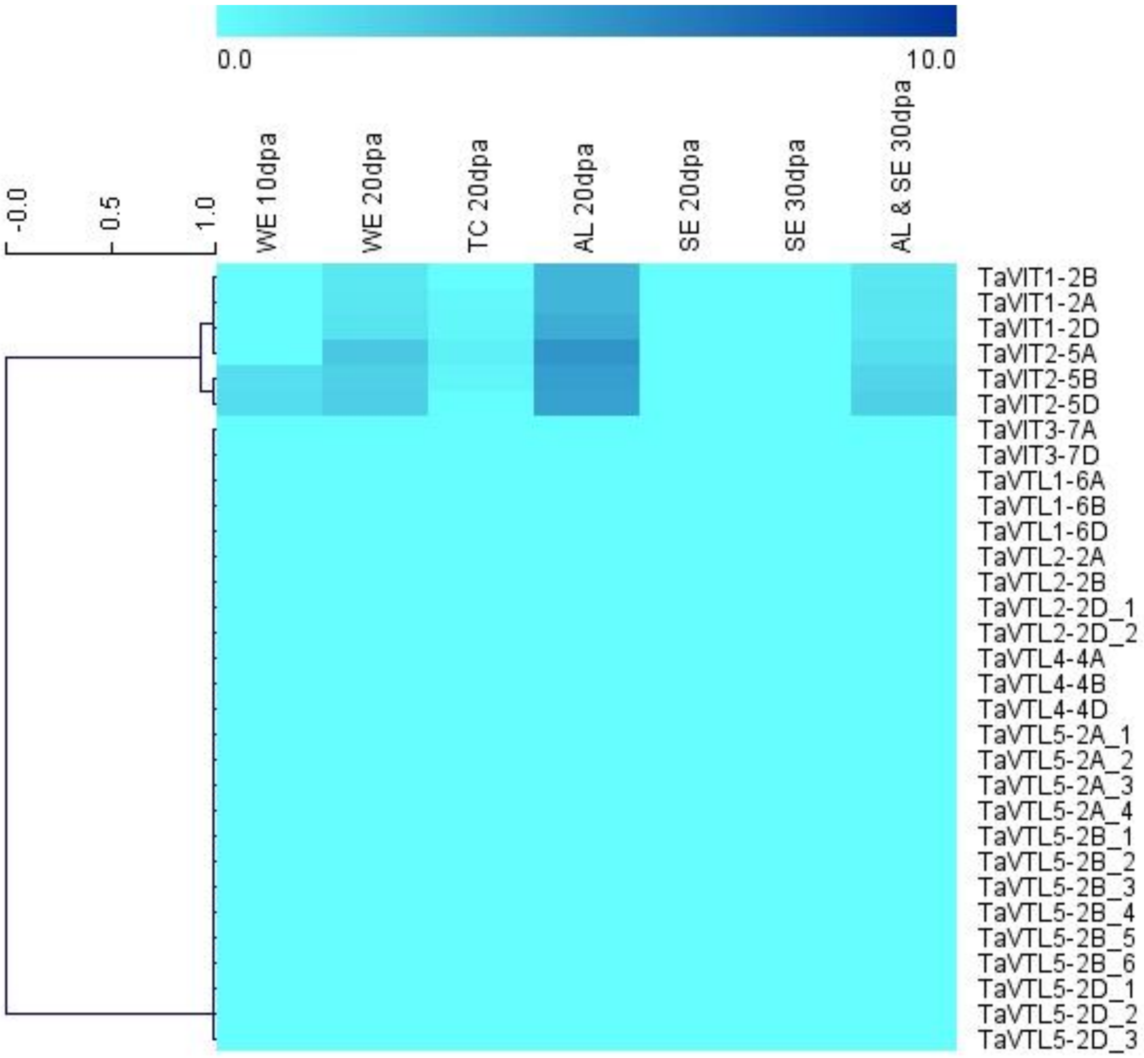

Figure S5. Heatmap depicting the expression of VIT family genes (VIT and VTL genes) in Grain Tissue Developmental Time-course (WE: Whole endosperm, TC: Transfer cells, AL: Aleurone layer, SE: Starchy Endosperm, dpa: days post anthesis). FPKM values were extracted using expVIP database. Increasing intensity of blue color shows increase in expression as shown by the color bar above.

Table 1. List of 31 VIT family genes extracted from ensembl biomart using Pfam ID: PF01988.

VIT family gene IDs	Peptide Length
TraesCS7A02G420900	218
TraesCS2D02G326300	248
TraesCS2A02G433900	210
TraesCS4D02G110600	221
TraesCS2B02G455000	182
TraesCS2D02G588000	192
TraesCS2D02G384100	231
TraesCS2A02G336600	246
TraesCS2D02G431900	210
TraesCS2A02G387100	236
TraesCS6A02G238800	232
TraesCS2B02G455200	210
TraesCS2B02G345300	246
TraesCS2A02G434000	210
TraesCS4A02G209600	199
TraesCS2B02G404700	236
TraesCS5A02G203400	245
TraesCS2A02G434100	125
TraesCS2B02G610400	206
TraesCS5B02G202100	245
TraesCS2B02G455100	210
TraesCS5D02G209900	256
TraesCS6B02G285700	231
TraesCS4B02G112900	269
TraesCS2A02G434200	210
TraesCS2D02G383900	231
TraesCS2B02G454900	212
TraesCS2D02G432000	212
TraesCS7D02G413000	245
TraesCS2B02G455300	212
TraesCS6D02G221100	231

Green: VTL; genes; Blue: VIT genes.

Table 2. List of VIT gene family (VTL and VIT genes) identified in wheat along with their gene ID, length of CDS and protein of the corresponding TaVTL and TaVIT proteins, number of exons-introns, subcellular localization and transmembrane domain related information.

Gene Name	ID	TM Domains (Phobius)	TM Domains (TMHMM)	Subcellular Localization (WolfPsort)	Peptide Length	CDS Length	Introns	Exons
**TaVIT1-2A**	TraesCS2A02G336600	4	3	plas: 11, vacu: 2, chlo: 1	246	741	3	4
**TaVIT1-2B**	TraesCS2B02G345300	4	4	plas: 11, vacu: 2, chlo: 1	246	741	3	4
**TaVIT1-2D**	TraesCS2D02G326300	4	4	plas: 11, vacu: 2, chlo: 1	248	747	3	4
**TaVIT2-5A**	TraesCS5A02G203400	4	5	chlo: 6, plas: 3, E.R.: 3, mito: 1, vacu: 1	245	738	3	4
**TaVIT2-5B**	TraesCS5B02G202100	4	4	chlo: 7, plas: 3, E.R.: 3, vacu: 1	245	738	3	4
**TaVIT2-5D**	TraesCS5D02G209900	4	4	chlo: 7, plas: 3, E.R.: 2, mito: 1, vacu: 1	256	738	3	4
**TaVIT3-7A**	TraesCS7A02G420900	3	3	plas: 10, vacu: 4	218	657	3	4
**TaVIT3-7D**	TraesCS7D02G413000	4	4	plas: 10, vacu: 2, chlo: 1, E.R.: 1	245	738	3	4
**TaVTL1-6A**	TraesCS6A02G238800	5	5	cyto: 4, E.R.: 3.5, E.R._plas: 3, mito: 2, plas: 1.5, extr: 1, vacu: 1, golg: 1	232	699	0	1
**TaVTL1-6B**	TraesCS6B02G285700	5	5	vacu: 13, plas: 1	231	696	0	1
**TaVTL1-6D**	TraesCS6D02G221100	5	5	vacu: 10, golg: 2, plas: 1, extr: 1	231	696	0	1
**TaVTL2-2A**	TraesCS2A02G387100	5	5	vacu: 13, plas: 1	236	711	0	1
**TaVTL2-2B**	TraesCS2B02G404700	5	5	vacu: 10, plas: 3, E.R.: 1	236	711	0	1
**TaVTL2-2D_1**	TraesCS2D02G383900	5	5	vacu: 13, plas: 1	231	696	0	1
**TaVTL2-2D_2**	TraesCS2D02G384100	5	5	vacu: 12, plas: 1, E.R.: 1	231	696	0	1
**TaVTL4-4A**	TraesCS4A02G209600	5	4	plas: 6.5, cyto_plas: 4, E.R.: 3, vacu: 2, extr: 1, golg: 1	199	600	0	1
**TaVTL4-4B**	TraesCS4B02G112900	6	6	E.R.: 5, plas: 4, golg: 3, chlo: 1, vacu: 1	269	810	0	1
**TaVTL4-4D**	TraesCS4D02G110600	5	5	vacu: 9, plas: 3, cyto: 1, E.R.: 1	221	666	0	1
**TaVTL5-2A_1**	TraesCS2A02G434000	3	3	vacu: 12, plas: 1, extr: 1	210	633	0	1
**TaVTL5-2A_2**	TraesCS2A02G434200	3	3	vacu: 11, golg: 2, plas: 1	210	633	0	1
**TaVTL5-2A_3**	TraesCS2A02G434100	3	3	vacu: 7, plas: 5, chlo: 1, E.R.: 1	125	378	0	1
**TaVTL5-2A_4**	TraesCS2A02G433900	3	3	vacu: 11, golg: 2, plas: 1	210	633	0	1
**TaVTL5-2B_1**	TraesCS2B02G455100	3	3	vacu: 12, plas: 1, extr: 1	210	633	0	1
**TaVTL5-2B_2**	TraesCS2B02G455200	3	3	vacu: 12, plas: 1, extr: 1	210	633	0	1
**TaVTL5-2B_3**	TraesCS2B02G454900	2	2	vacu: 12, plas: 1, extr: 1	212	639	0	1
**TaVTL5-2B_4**	TraesCS2B02G455300	3	3	vacu: 11, golg: 2, plas: 1	212	639	0	1
**TaVTL5-2B_5**	TraesCS2B02G455000	2	3	vacu: 12, plas: 1, extr: 1	182	549	0	1
**TaVTL5-2B_6**	TraesCS2B02G610400	4	4	vacu: 11, plas: 2, golg: 1	206	621	0	1
**TaVTL5-2D_1**	TraesCS2D02G432000	3	4	vacu: 12, plas: 1, extr: 1	212	639	0	1
**TaVTL5-2D_2**	TraesCS2D02G431900	3	3	vacu: 12, plas: 1, extr: 1	210	633	0	1
**TaVTL5-2D_3**	TraesCS2D02G588000	5	5	vacu: 12, plas: 1, golg: 1	192	579	0	1

Gray cells: Predicted to have a possible N-terminal signal peptide.

Table S3. Conserved motifs identified in VTL and VIT proteins using MEME suite. The color code, consensus sequence logo, E-value and the number of proteins in which each motif was found are listed in the table.

Motif No.	Color code	Motif Logo	E-value	No. of Sequences Containing Motif
Motif 1		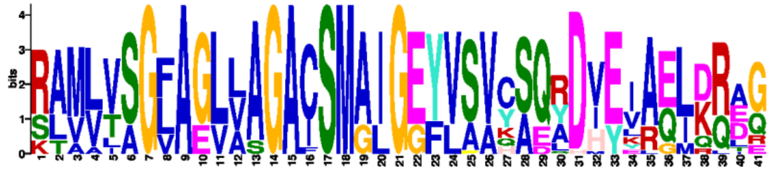	1.7e−642	29
Motif 2		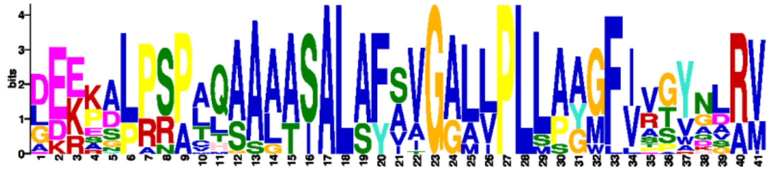	4.7e−569	31
Motif 3		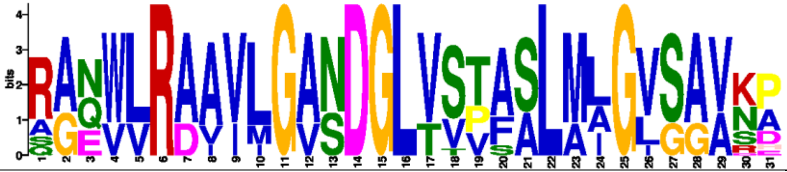	5.9e−477	30
Motif 4		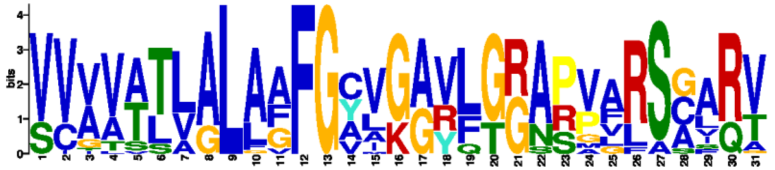	5.4e−353	30
Motif 5		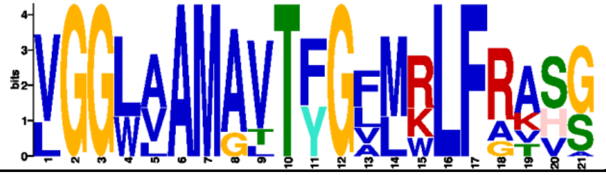	5.50E−209	21

Motif 6		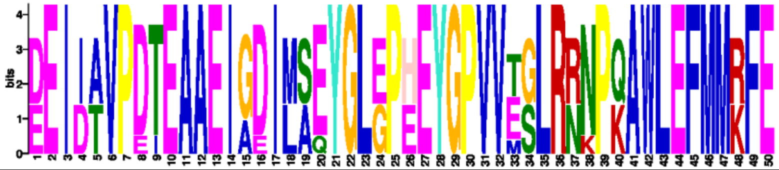	9.40E−207	7
Motif 7		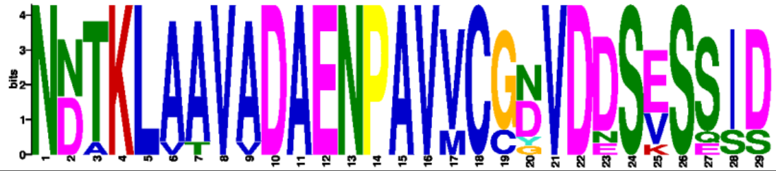	3.30E−160	10
Motif 8		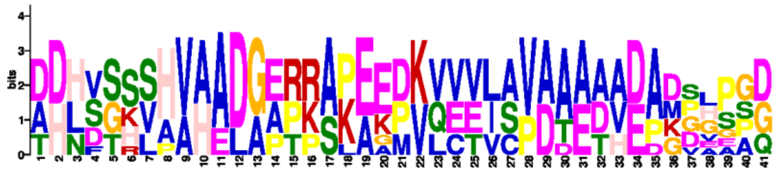	1.10E−59	9
Motif 9		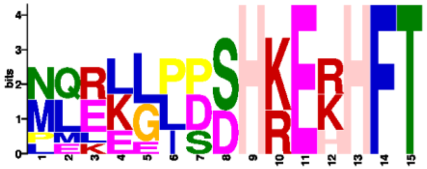	3.40E−49	8
Motif 10		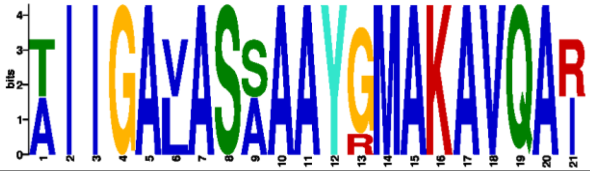	6.00E−33	6
Motif 11		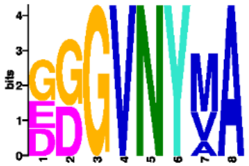	1.30E−05	7

Motif 12		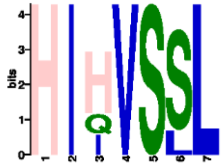	3.80E−05	5
Motif 13		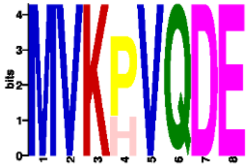	7.10E−03	3
Motif 14		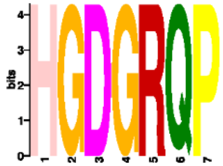	4.00E−02	3

Table S4. Metal-responsive cis-elements found in VTL and VIT gene promoter regions.

Gene Name	ID	cis-elements			
		IDE1	HMRE	MRE	IRO2
**TaVIT1-2A**	TraesCS2A02G336600	−1209 to −1213	-	-	-
**TaVIT1-2B**	TraesCS2B02G345300	−632 to −636	-	-	-
**TaVIT1-2D**	TraesCS2D02G326300	−442 to −446	−1353 to −1359	-	-
**TaVIT2-5A**	TraesCS5A02G203400	−1002 to −1006	-	-	-
		−957 to −961	-	-	-
**TaVIT2-5B**	TraesCS5B02G202100	−946 to −950	−883 to −889	-	-
		−893 to −897	-	-	-
		−840 to −844	-	-	-
**TaVIT2-5D**	TraesCS5D02G209900	−1023 to −1027	−960 to −966	-	-
		−970 to −974	-	-	-
		−927 to −931	-	-	-
**TaVIT3-7A**	TraesCS7A02G420900	−696 to −700	-		
		−387 to −391	-	-	-
**TaVIT3-7D**	TraesCS7D02G413000	−1483 to −1487	−1267 to −1273	-	-
		−1356 to −1360	-	-	-
		−1244 to −1248	-	-	-
		−1201 to −1205	-	-	-
**TaVTL1-6A**	TraesCS6A02G238800	−213 to −217	−703 to −709	−125 to −134	
		-	−50 to −56	-	-
**TaVTL1-6B**	TraesCS6B02G285700	-	−47 to −53	-	-
**TaVTL1-6D**	TraesCS6D02G221100	−282 to −286	−50 to −56	−1081 to −1090	-
**TaVTL2-2A**	TraesCS2A02G387100	−1203 to −1207	−1325 to −1331	-	-
		−794 to −798	-	-	-
**TaVTL2-2B**	TraesCS2B02G404700	−265 to −269	-	-	−1056 to −1063
**TaVTL2-2D_1**	TraesCS2D02G383900	−500 to −504	-	-	−1089 to −1096
		−246 to −250	-	-	−1452 to −1459
**TaVTL2-2D_2**	TraesCS2D02G384100	−501 to −505	-	-	−1086 to −1093
		−243 to −247	-	-	−1049 to −1056
**TaVTL4-4A**	TraesCS4A02G209600	−935 to −939	−1448 to −1454	-	-
		−714 to −718	−1386 to −1392	-	-
**TaVTL4-4B**	TraesCS4B02G112900	-	-	-	-
		-	-	-	-
**TaVTL4-4D**	TraesCS4D02G110600	-	-	−427 to −436	-
**TaVTL5-2A_1**	TraesCS2A02G434000	−1482 to −1486	-	−901 to −910	-
		−877 to −881	-	-	-
**TaVTL5-2A_2**	TraesCS2A02G434200	−1355 to −1359	-	-	-
		−1351 to −1355	-	-	-
		−735 to −739	-	-	-
		−709 to −713	-	-	-
		−140 to −144	-	-	-
**TaVTL5-2A_3**	TraesCS2A02G434100	−127 to −131	−1307 to −1313	−1104 to −1113	-
		−23 to −27	-	-	-
**TaVTL5-2A_4**	TraesCS2A02G433900		−1456 to −1462	−1253 to −1262	-
**TaVTL5-2B_1**	TraesCS2B02G455100	−737 to −741	-	-	-
**TaVTL5-2B_2**	TraesCS2B02G455200	−737 to −741	-	-	-
**TaVTL5-2B_3**	TraesCS2B02G454900	−730 to −734	-	-	-
		−610 to −614	-	-	-
**TaVTL5-2B_4**	TraesCS2B02G455300	−139 to −143	-	-	-
**TaVTL5-2B_5**	TraesCS2B02G455000	−1307 to −1311	-	-	-
		−738 to −742	-	-	-
**TaVTL5-2B_6**	TraesCS2B02G610400	−769 to −773	-	-	-
		−473 to −477	-	-	-
		−164 to −168	-	-	-

**TaVTL5-2D_1**	TraesCS2D02G432000	−1436 to −1440	-	-	-
		−1432 to −1436	-	-	-
		−638 to −642	-	-	-
		−612 to −616	-	-	-
		−139 to −143	-	-	-
**TaVTL5-2D_2**	TraesCS2D02G431900	−1337 to −1341	-	-	-
		−1056 to −1060	-	-	-
		−726 to −730	-	-	-
**TaVTL5-2D_3**	TraesCS2D02G588000	−837 to −841	-	-	-
		−543 to −547	-	-	-
		−208 to −212	-	-	-

Table 5. List of gene specific primers used for qRT-PCR for *TaVTL genes.*

Gene name	Amplicon size	Primer sequence (5′–3′)
TaVTL5 F	151 bp	AGCTGGACCAGGCCGGAAAG
TaVTL5 R	151 bp	CACGACGACCACCACGGC
TaVTL1 F	120 bp	TCATGATCGGCGTCGGCGCC
TaVTL1 R	120 bp	CGGCGGGTGCGCTTGATCTG
TaVTL4 F	179 bp	ACCGACAATGACACCAAGCTCGCT
TaVTL4 R	179 bp	GACGGCGGCGCGCAGCCAC
TaVTL2 F	203 bp	ACATGGCCCGCGCGCAGTGG
TaVTL 2 R	203 bp	GATGTCGTACTGCGCGTACACGG
